# Osteocyte-Derived Insulin-Like Growth Factor I Is Not Essential for the Bone Repletion Response in Mice

**DOI:** 10.1371/journal.pone.0115897

**Published:** 2015-01-30

**Authors:** Kin-Hing William Lau, David J. Baylink, Matilda H.-C. Sheng

**Affiliations:** 1 Division of Regenerative Medicine, Department of Medicine, Loma Linda University School of Medicine, Loma Linda, California, United States of America; 2 Musculoskeletal Disease Center, Jerry L. Pettis Memorial VA Medical Center, Loma Linda, California, United States of America; Inserm U606 and University Paris Diderot, FRANCE

## Abstract

The present study sought to evaluate the functional role of osteocyte-derived IGF-I in the bone repletion process by determining whether deficient expression of *Igf1* in osteocytes would impair the bone repletion response to one week of dietary calcium repletion after two weeks of dietary calcium deprivation. As expected, the two-week dietary calcium depletion led to hypocalcemia, secondary hyperparathyroidism, and increases in bone resorption and bone loss in both *Igf1* osteocyte conditional knockout (cKO) mutants and WT control mice. Thus, conditional disruption of *Igf1* in osteocytes did not impair the calcium depletion-induced bone resorption. After one week of calcium repletion, both cKO mutants and WT littermates showed an increase in endosteal bone formation attended by the reduction in osteoclast number, indicating that deficient *Igf1* expression in osteocytes also did not have deleterious effects on the bone repletion response. The lack of an effect of deficient osteocyte-derived IGF-I expression on bone repletion is unexpected since previous studies show that these *Igf1* osteocyte cKO mice exhibited impaired developmental growth and displayed complete resistance to bone anabolic effects of loading. These studies suggest that there is a dichotomy between the mechanisms necessary for anabolic responses to mechanical loading and the regulatory hormonal and anabolic skeletal repletion following low dietary calcium challenge. In conclusion, to our knowledge this study has demonstrated for the first time that osteocyte-derived IGF-I, which is essential for anabolic bone response to mechanical loading, is not a key regulatory factor for bone repletion after a low calcium challenge.

## Introduction

Mammalian bones have two remarkable properties that are essential for the maintenance of mechanical integrity of the skeleton: First, they constantly replace damaged or fatigued bony tissues with new and mechanically sound bone through a cellular process known as the bone remodeling. Bone remodeling is accomplished through collaborative and sequential actions of osteoclasts and osteoblasts to mediate bone resorption and formation, respectively, in a coupled manner [1]. Accordingly, the increase in bone resorption is followed by the increase in compensatory bone formation of equal magnitude, such that there is no loss in bone mass or mechanical integrity as the result of bone remodeling. Second, mammalian bones can adjust their shape and size to adapt to changes in mechanical stresses by removing bone mass from bone sites of reducing mechanical load and promoting bone formation at sites where mechanical stress is increased [2]. Contrary to the coupled bone formation in bone remodeling, the loading-induced bone formation is not spatially or temporally coupled to resorption [2].

In addition to providing mechanical supports and protection for internal organs, mammalian bones also serve as the skeletal reservoir of essential bone minerals [3]. When there is an urgent bodily need for bone minerals (e.g., dietary calcium insufficiency or lactation), bone resorption is increased to release bone minerals to maintain bone mineral homeostasis. When the demand dissipates (e.g., dietary calcium repletion or weanling), the lost bone mass is rapidly regenerated through a process known as the bone repletion. The bone repletion is the guardian of bone maintenance and is an important bone repair mechanism to restore bone mass in time when the coupling process of bone formation to resorption is subverted [4,5]. Defective bone repletion would eventually lead to osteoporosis and an increased fracture risk [6]. Bone depletion/repletion process is mechanistically similar to bone remodeling [4], with the exception that the bone repletion process is spatially or temporally uncoupled to the bone depletion and it occurs only after removal of the mineral stress. The bone repletion, along with the osteogenic response to loading and the healing of fractures, are the three major physiological bone regenerative processes. Thus, a better understanding of the bone repletion process is essential for our overall understanding, not only of the pathophysiology of osteoporosis, but also of the mechanism(s) regulating bone regeneration.

Recent studies have implicated an important regulatory role for osteocytes in the bone formation response to mechanical loading and in bone remodeling [7,8,9]. The osteocyte has an extensive network of dendrites extending to and making contact with other osteocytes, periosteal and endosteal lining cells, bone surface osteoblasts and osteoclasts, and bone marrow cells through an interconnected, widespread, canaliculi system [10]. This network also allows soluble paracrine signaling molecules migrate freely from the osteocyte to “communicate” with other bone cells. Thus, osteocytes are ideally positioned in the bone to sense mechanical and biochemical signals to regulate the loading-induced bone formation and bone remodeling [11]. The mechanism of the osteocyte regulation of bone remodeling likely involves osteocyte-derived signaling mediators. Osteocytes have been shown to release paracrine factors, such as sclerostin (SOST) [12] and receptor activator of nuclear factor-κ B ligand (RANKL) [13,14], to locally regulate bone formation and resorption, respectively. Osteocytes also secrete endocrine effectors, such as fibroblast growth factor-23 (FGF-23), to regulate phosphate and vitamin D homeostasis in the kidneys [15,16]. We are interested in osteocyte-derived insulin-like growth factor (IGF)-I as the soluble osteocyte-derived signaling factor for the following reasons: 1) Bone is a major target organ for IGF-I [17,18], which not only promotes bone formation and developmental bone growth [19,20] but also enhances bone resorption [21]; 2) IGF-I is a critical signaling mediator of the bone formation response to PTH [22] and a key mediator of the PTH/PTHrP receptor signaling in the osteocyte regulation of periosteal bone formation and intracortical remodeling [23,24]; and 3) osteocytes express substantial amounts of IGF-I [25]; and conditional disruption of the *Igf1* gene in osteocytes impaired developmental bone growth that was associated with suppression in bone turnover [26]. Our interest in osteocyte-derived IGF-I is further enhanced by the finding that osteocyte-derived IGF-I is an essential mediator of the osteogenic response to loading [27]. We envisioned that osteocyte-derived IGF-I could also be a key regulatory mediator in bone remodeling, especially in the bone repletion process.

The present study sought to test the hypothesis that deficient expression of *Igf1* in osteocytes would impair the bone repletion process using a mouse dietary calcium depletion/repletion model. The calcium depletion-repletion model was used, because: 1) dietary calcium restriction triggers increases in bone resorption along with suppression of bone formation and mineralization [28]; 2) dietary calcium repletion rapidly regenerates the bone mass that was lost during calcium depletion [29]; and 3) the increased bone formation upon dietary calcium repletion is one of the most physiologically relevant bone repletion processes.

## Methods

### Animals

**Figure 1:**
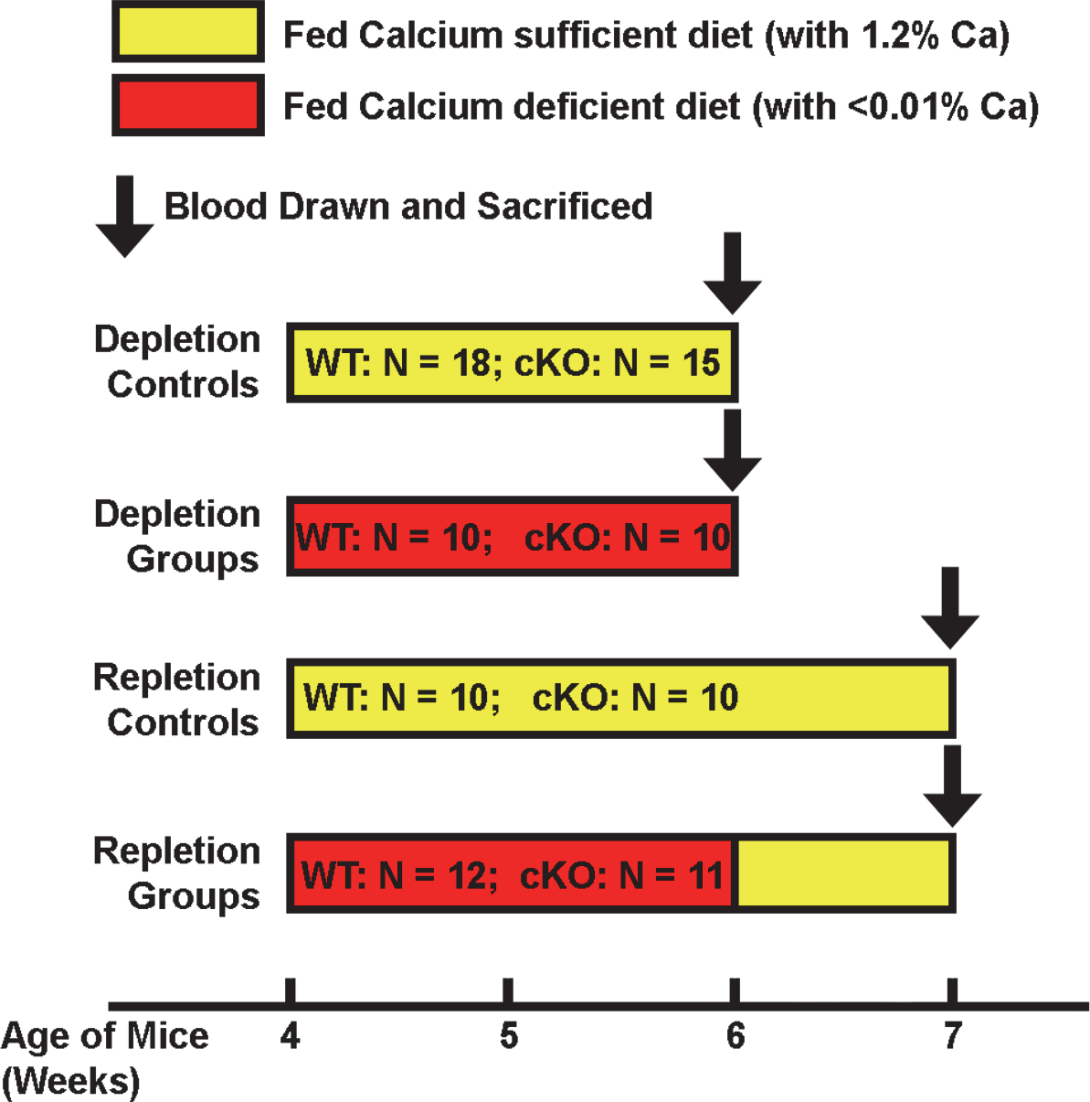
Schematic representation of the experimental design of the dietary calcium depletion and repletion experiment. The indicated numbers of 4-week-old female *Igf1* osteocytic cKO mutants and age- and gender-matched WT littermates were each divided into four groups: 1) depletion control groups, which were fed the calcium sufficient diet (with 1.2% calcium) for 2 weeks; 2) depletion experimental groups, which were fed the calcium deficient diet (with <0.01% calcium) for the same 2 weeks; 3) repletion control groups, which were fed the calcium sufficient diet for 3 weeks; and 4) repletion experimental groups, which were fed the calcium deficient diet for two weeks followed by the calcium sufficient diet for one week. At the end of each regimen, all mice were sacrificed and blood drawn for measurements of plasma parameters. Femurs were isolated, cleaned, fixed with formalin and subjected pQCT and histomorphometry measurements. Tibias were isolated, and total RNA was isolated for real-time RT-PCR measurements.

The osteocyte *Igf1* conditional knockout (cKO) mice were generated, as previously described [26], by crossbreeding *Igf1* ^loxp/loxp^ transgenic mice with DMP1-*Cre* transgenic mice, in which the *Cre* transgene was driven by the 9.6-kb DMP1 promoter. Four-week-old female osteocyte *Igf1* cKO mice and WT littermates were used for this work. Calcium deficiency was created through dietary calcium deprivation by subjecting mice for two weeks to a diet with low (< 0.01%) calcium content (Harlan, Madison, WI). Weanling mice were used, because previous studies have shown that weanling rodents are highly susceptible to the calcium depletion stress with large decreases in bone mass along with corresponding changes in bone resorption and formation parameters [4,5,28,30,31,32,33,34,35]. The experimental design of this calcium depletion/repletion protocol is shown in Fig. 1. A group of mice of each genotype were fed a diet containing 1.2% calcium (Harlan) in parallel as the control group for depletion. To evaluate the effect of dietary calcium repletion, a separate group of cKO mice and corresponding WT littermates were fed the diet containing 1.2% calcium for up to 7 days immediately after the two-week of dietary calcium depletion. Another group of mice of each genotype was fed a diet containing 1.2% calcium throughout the entire three weeks of the experiment as repletion control groups. All animals had free access to water and food, and were housed in groups of 4 mice per cage in a 12-hr light/12-hr dark cycle. All animal use protocols were approved by the Institutional Animal Care and Use Committee (IACUC) of Loma Linda University and also by the Animal Care and Use Review Office (ACURO) of the United States Department of Army.

### Plasma biomarkers of bone turnover

Blood was drawn at noon of the day of sacrifice. Briefly, animals were euthanized by inhalation of an overdose of carbon dioxide. Blood was collected through cardiac puncture using a 25-G needle into heparinized tubes. Plasma was prepared by low-speed centrifugation (3,000 × g for 15 min at 4°C), and each plasma sample was stored in aliquots at −80°C until assay. Plasma levels of c-terminal telopeptide of type I collagen (CTx), procollagen type I N-terminal propeptide (P1NP), FGF-23, and PTH_(1–84)_ were measured with respective Enzyme-Linked ImmunoSorbent Assay (ELISA) kit; purchased from either Immunodiagnostics Systems (Fountain Hills, AZ) or Immunoptics (San Clemente, CA). Plasma calcium and phosphorus levels were determined with commercial colorimetric assays obtained from Biovision (Milpitas, CA). All assays were performed according to instruction provided by the respective supplier. The color intensity of the product in each ELISA was analyzed by a Synergy HT Biotek microplate reader with the Gen5 software (BioTek Instruments, Winooski, CT).

### Quantitative RT-PCR

After removal of muscles and connective tissue layers, tibias were frozen at −80°C until extraction for total RNA. Briefly, tibias (without flushing out the bone marrow) were pulverized in liquid nitrogen. Total RNA was isolated from bone powders using the Qiazol Lysis Reagent and purified with RNeasy Mini kit (Qiagen, Valencia, CA). The quality of each RNA sample was assessed with Agilent 2100 Bioalanyzer (Agilent Technologies Inc., Santa Clara, CA) using the RNA 6000 nano-Kit (Agilent Technologies Inc.). Only RNA samples with high quality of RNA were used in subsequent RT-PCR assays. The cDNA was generated from each RNA sample by reverse transcription using the ThermoScript RT kit (Invitrogen, La Jolla). The relative level of each gene-of-interest was determined with the SyBr Green PCR kit (Applied Biosystems) in an ABI 7500 Fast cycler (Applied Biosystems, Foster City, CA). The PCR primer set sequences of each gene-of-interest are shown in Table 1.

**Table 1:** Sequence of PCR primer sets for the test mouse genes.

***Gene***	***Accession Number***	***Forward Primer***	***Reverse Primer***
*Actin*	NM_007393	5’caggcattgctgacagga	5’tgctgatccacatctgctgg
*Opg*	MMU94331	5’cacacacactggggactctg	5’cagctgtgaggagaggaagg
*Rankl*	AF019048.1	5’cacagccctctctcttgagc	5’gactgtgacccccttccata
*Sost*	NM_024449	5’tcaggaatgatgccacagag	5’tgtcaggaagcgggtgtagt

### pQCT analysis

Femurs after dissection were fixed in 10% formalin overnight and were stored in phosphate buffered saline (PBS) at 4°C until analyses. The length of each femur was measured with a digital caliper. pQCT measurements were performed with the STRATEC XCT 960M (Norland Medical Systems, Ft. Akinson, WI) and analyzed with the version 6.00 software program provided by the manufacturer as described previously [26]. Briefly, two slices per femur at the position of 20% and 50% of the total femur length from the distal end, which corresponded to metaphysis and mid-point of the femur shaft, respectively, were scanned. The inner and outer thresholds were set at 230 and 630 mg/cm^3^, respectively, to distinguish the cortical compartment from the trabecular compartment of bones of mice fed a normal calcium-containing diet. To adjust for the reduced bone mineral density (BMD) in bone of mice that fed the calcium-deficient diet, the inner and outer thresholds were re-set at 180 and 400 mg/cm^3^, respectively.

### Bone histomorphometry

Static bone histomorphometry of femurs was determined as previously described [36]. Trabecular bone parameters were measured at the secondary spongiosa of the femur. To determine endosteal bone formation parameters, mice were injected with 25 mg/kg body weight of demeclocycline and 25 mg/kg body weight of tetracycline at day 6 and day 1 prior to euthanasia (i.e., a 5 day inter-labeling time). Twenty four hours after the second injection, mice were euthanized and femurs were removed and immediately fixed in 10% formalin. Bone was subsequently dehydrated in ethanol, infiltrated, and embedded into methylmethacrylate (MMA). Longitudinal thin sections (7.5 μm in thickness) were obtained with the *Leica* Microtome (Leica Microsystems, Inc., Buffalo Grove, IL). A set of bone specimens was demineralized for 21 days in 14% EDTA solution, embedded into paraffin wax, and sectioned. All histomorphometric measurements was performed with an Olympus fluorescent microscope BX51 (Tokyo, Japan) using the Osteomeasure digitalized system (OsteoMetrics, Atlanta, GA). Total tetracycline labeled surface (L.Pm) was the sum of double labeled surface length (dL.Pm) and one half of the single labeled surface length (sL.Pm). The mineral apposition rate (MAR) was the distance of the double labels divided by the inter-labeling time (5 days). Bone formation rate (BFR) was calculated by multiplying MAR by L.Pm.

### Statistical Analysis

Results were shown as mean ± standard error of the mean (SEM). Each mouse strain (WT mice and cKO mutants) has two experimental groups (the depletion group and the repletion group) and two age- and gender-matched corresponding control groups. Statistical significance of the differences in each mouse strain was determined with two-tailed *Student*’s *t*-test by comparing the effect on the experimental group with that on corresponding control group. The individual effect of the genotype (WT vs. cKO) or the diet (normal vs. calcium-deficient diet) as well as the interaction between genotype and diet were determined on all four groups together by two-way analysis of variance (ANOVA). The difference was considered significant, when P< 0.05.

## Results

### Effects of deficient *Igf1* expression in osteocytes on plasma levels of bone minerals, PTH, and FGF-23 after two-week dietary calcium depletion

**Figure 2:**
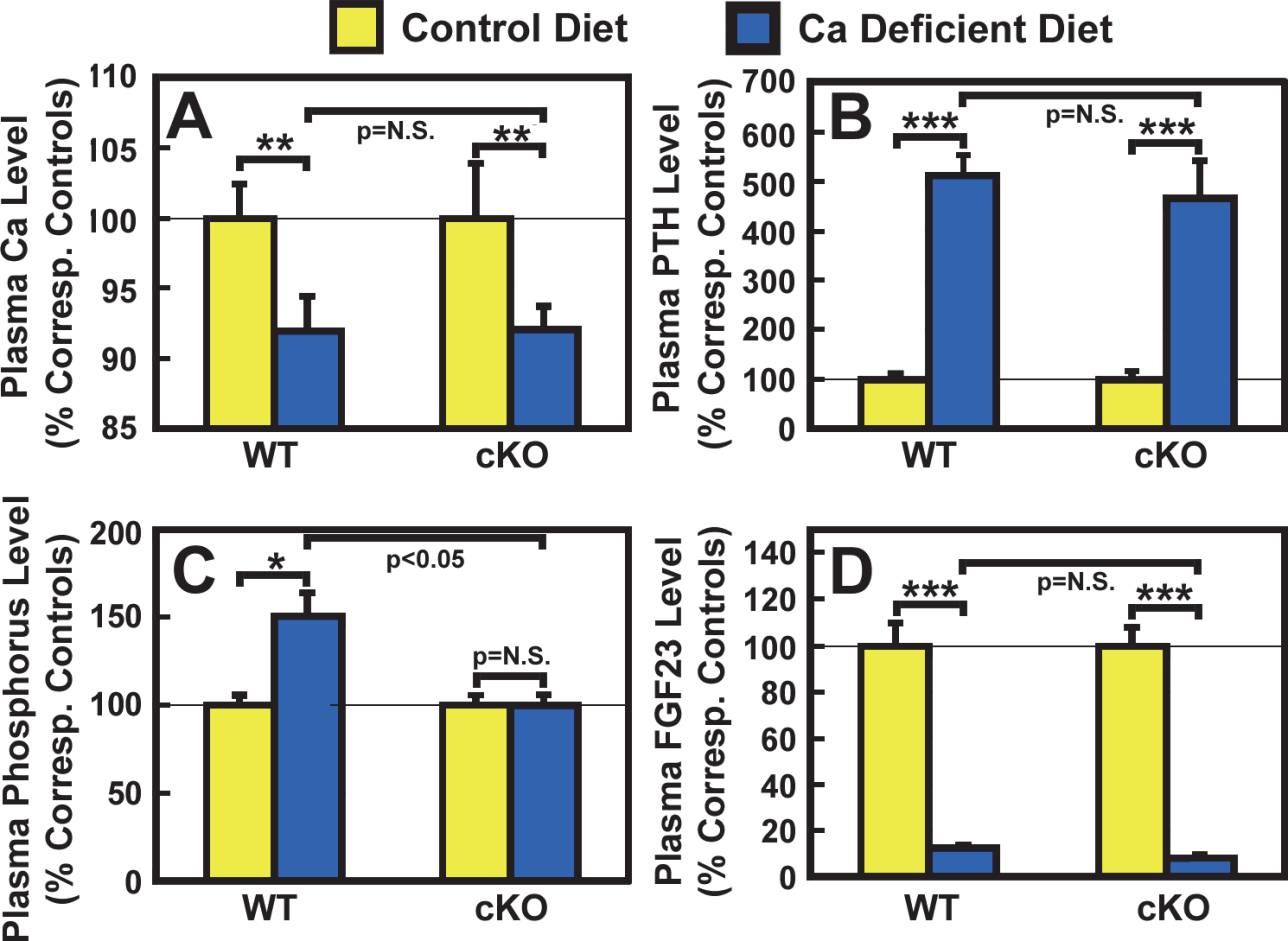
Effects of two-week dietary calcium restriction on plasma calcium (A), PTH (B), phosphorus (C), and FGF23 (D) of osteocyte *Igf1* cKO mice and those of WT mice. Four-week-old female osteocyte *Igf1* cKO mice and age- and gender-matched WT control mice were fed a mouse diet containing either <0.01% calcium (calcium-deficient diet) or 1.2% calcium (control diet) for two weeks. At the end of the calcium depletion, blood samples were collected from each mouse for measurements of calcium (Ca), PTH, phosphorus, and FGF23. Results are shown in relative percentage of the respective level of each metabolite in corresponding control mice that were fed the control calcium-containing diet (mean ± S.E.M). The number of animals in each test group is shown in Table 2. *P<0.05; **P<0.01; and ***P<0.001. P = N.S. (not significant), where P>0.05.

Under basal conditions (fed a diet containing sufficient amounts of calcium), plasma level of PTH, but not that of calcium or phosphorus, of osteocyte *Igf1* cKO mutant mice were significantly higher than those of corresponding WT control mice (Table 2). After two weeks of dietary calcium deprivation, the plasma calcium level of both WT control mice and cKO mutants was reduced significantly by 8% each (Fig. 2A), and was attended by the corresponding 4-fold increase in plasma PTH level over the basal level (Fig. 2B). These findings confirm the previous studies that rodents fed a low calcium diet at weanling developed hypocalcemia and secondary hyperparathyroidism [28,30,34,35] and indicate that osteocyte-derived IGF-I does not play a role in the development of hypocalcemia and secondary hyperparathyroidism in response to the calcium stress. The calcium deficiency increased plasma phosphorus level slightly but significantly (P<0.05) in WT mice, but not in cKO mutants (Fig. 2C). Consistent with a previous report that calcium deficiency reduced circulating levels of FGF-23 [37], the two-week dietary calcium restriction led to highly significant reduction in the plasma level of FGF-23 in WT control mice and in cKO mutants (Table 2). The reduction in plasma FGF-23 in both mouse strains was similar in the percentage of respective basal level, each by ~60% (Fig. 2D). These findings suggest that osteocyte-derived IGF-I also does not have a regulatory role in the calcium deficiency-mediated changes in circulating levels of phosphorus or FGF-23.

**Table 2:** Comparison of effects of the two-week dietary calcium depletion on plasma levels of Ca, P, PTH, and FGF-23 level in 4 weeks-old female osteocyte *Igf1* conditional knockout (cKO) mice with those in age- and sex-matched WT control mice.

**Plasma parameters**	**Calcium-containing Control Diet**		**Calcium Deficient Diet**		**Two-way ANOVA**		
	**WT control mice**	**cKO mice**	**WT control mice**	**cKO mice**	**Diet**	**Gene**	**Interaction**
Ca, mg/dl	12.5±0.3 (n = 10)	12.7±0.5 (n = 8)	11.5±0.3** (n = 12)	11.7±0.2** (n = 7)	P<0.01	P = N.S.	P = N.S.
PTH, pg/ml	38±4 (n = 8)	53±9^a^(n = 6)	194±16*** (n = 10)	247±39*** (n = 7)	P<0.01	P = N.S.	P = N.S.
Phosphorus, μM	7.3±0.4 (n = 14)	8.3±0.5 (n = 8)	11.0±1.0* (n = 8)	8.3±0.5 (n = 6)	P<0.01	P = N.S.	P<0.01
FGF-23, ng/ml	386±38 (n = 10)	438±34^a^(n = 4)	49±3*** (n = 5)	37±3*** (n = 5)	P<0.0001	P = N.S.	P = N.S.

^a^ P<0.05, when compared to WT control mice on basal calcium-containing diet.

* P<0.05, ** P<0.01; and ***P<0.001, compared to corresponding controls on calcium-containing diet of each respective mouse strain.

P = N.S. (not significant), when P>0.05.

### Effects of deficiency in osteocyte-derived IGF-I on the calcium deficiency-induced resorption

**Figure 3:**
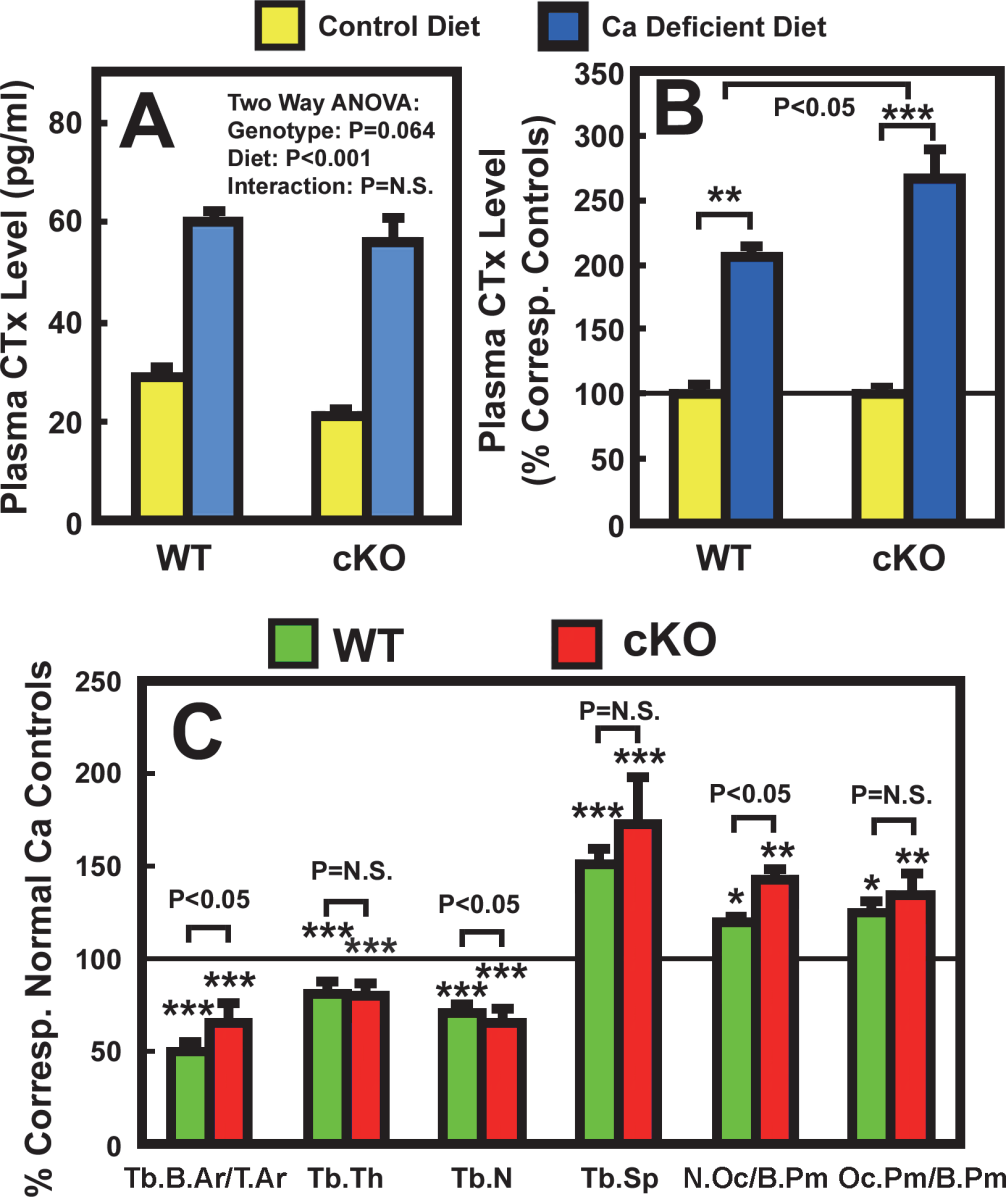
Effects of two-week dietary calcium restriction on plasma CTx (A and (B) and on histomorphometric parameters of the femur (C) of osteocyte *Igf1* cKO mice and those of WT mice. A: The same plasma samples used in Fig. 1 were assayed for CTx level. The left panel shows the actual CTx levels, and the right panel shows the relative percentage of the corresponding basal CTx levels. The number of animals in each group is between 8 and 10. B: static bone histomorphometric parameters of the femur bone of each mouse. The number of mice per group is shown in Table 3. Results are shown as mean ± S.E.M. in relative percentage of the respective level of each metabolite in corresponding control mice that were fed the calcium-containing control diet throughout the study. *P<0.05; **P<0.01; and ***P<0.001. P = N.S. (not significant), where P>0.05.

We next evaluated the potential mediating role of osteocyte-derived IGF-I in the calcium deficiency-induced bone resorption. We first compared the plasma level of CTx (a bone resorption biomarker) in osteocyte *Igf1* cKO mutants with that in age- and gender-matched WT control mice. Despite a 40% higher basal plasma PTH level in cKO mutants (Table 2), the basal plasma CTx level in cKO mutants was significantly lower by ~28% (P<0.05) than that in WT control mice (Fig. 3A), suggesting the higher basal PTH level in cKO mutants did not lead to an elevated basal bone resorption. However, the two-week calcium depletion significantly increased plasma CTx level in both cKO mutants and WT control mice (Table 2). When the plasma CTx level was normalized against respective basal level in each mouse strain (Fig. 3B), the calcium depletion-induced increase in plasma CTx in cKO mutants was significantly greater than that in WT mice (164±14% increase over basal in cKO mice vs. 109±5% increase over basal in WT mice, P<0.05). The possibility that deficient expression of *Igf1* in osteocytes may promote calcium deficiency-induced bone resorption is further supported by the marginally significant (P = 0.064) genotype effect (Fig. 3A).

**Table 3:** Effects of the two-week dietary calcium depletion on static bone histomorphometric parameters of trabecular bone at the secondary spongiosia of the distal femur.

	**WT controls**		**Osteocyte *Igf1* cKO mutants**		**Two-way ANOVA**		
	**Calcium-containing diet (n = 7)**	**Calcium-deficient diet (n = 7)**	**Calcium-containing diet (n = 6)**	**Calcium-deficient diet (n = 6)**	**Diet**	**Gene**	**Interaction**
Tb.B.Ar/T.Ar (%)	21.8±2.8	11.1±1.0^a^	14.4±1.4	9.4±1.6^a^	P<0.01	P = N.S.	P = N.S.
Tb.Wi (μm)	41.5±3.6	33.8±2.4^a^	41.3±1.5	33.2±2.5^a^	P<0.01	P = N.S.	P = N.S.
Tb.N (#)	4.56±0.44	3.25±0.17^a^	4.23±0.28	2.77±0.31^a^	P<0.01	P = N.S.	P = N.S.
Tb.Sp (μm)	185±23	279±17^a^	205±17	355±51^a^	P<0.01	P = N.S.	P = N.S.
N.Oc/B.Pm (#/μm^2^)	8.1±0.6	9.7±0.3	7.9±0.8	11.3±0.4^b^	P<0.05	P = N.S.	P = N.S.
Oc.Pm/B.Pm (%)	23.0±2.9	28.9±1.4^c^	19.8±1.7	26.7±2.2^a^	P<0.05	P = N.S.	P = N.S.

^a^ P<0.001; ^b^ P<0.01; and ^c^ P<0.05, when compared to corresponding control mice of each respective mouse strain that were fed the basal calcium-containing diet.

P = N.S. (not significant), when P>0.05.

We next compared several bone resorption-related static histomorphometric parameters of trabecular bone between the two mouse strains. As previously reported [26], there were no significant differences in trabecular bone area (Tb.B.Ar/T.Ar), number (Tb.N), thickness (Tb.Wi), and separation (Tb.Sp) between WT mice and cKO mutants under basal conditions (Table 3). The number of osteoclasts per bone surface (N.Oc/B.Pm) and the percentage of TRAP positive bone length per bone surface (Oc.Pm/B.Pm)] in cKO mutants under basal conditions were slightly (by 2.5% and 14%, respectively) but not significantly lower than those in WT control mice (Table 3). The dietary calcium depletion reduced Tb.N and Tb.Wi, resulting in reduction in Tb.B.Ar/T.Ar and increase in Tb.Sp in both mouse strains. The Oc.Pm/B.Pm was also increased in response to calcium depletion in both mouse strains (Table 3). When the calcium depletion-induced changes in these parameters were normalized against corresponding basal level, the reduction in Tb.N and the increase in N.Oc/B.Pm in cKO mutants were each significantly greater than those in WT mice (Fig. 3C). The increases in Tb.Sp and in Oc.Pm/B.Pm in the cKO mutants were also larger, albeit not significant statistically, than those in WT mice. These findings, along with the larger increase in plasma CTx (Fig. 3B), are consistent with the interpretation that conditional disruption of *Igf1* gene in osteocytes could have led to a small but significant enhancement in the bone resorption response to calcium deficiency.

**Figure 4:**
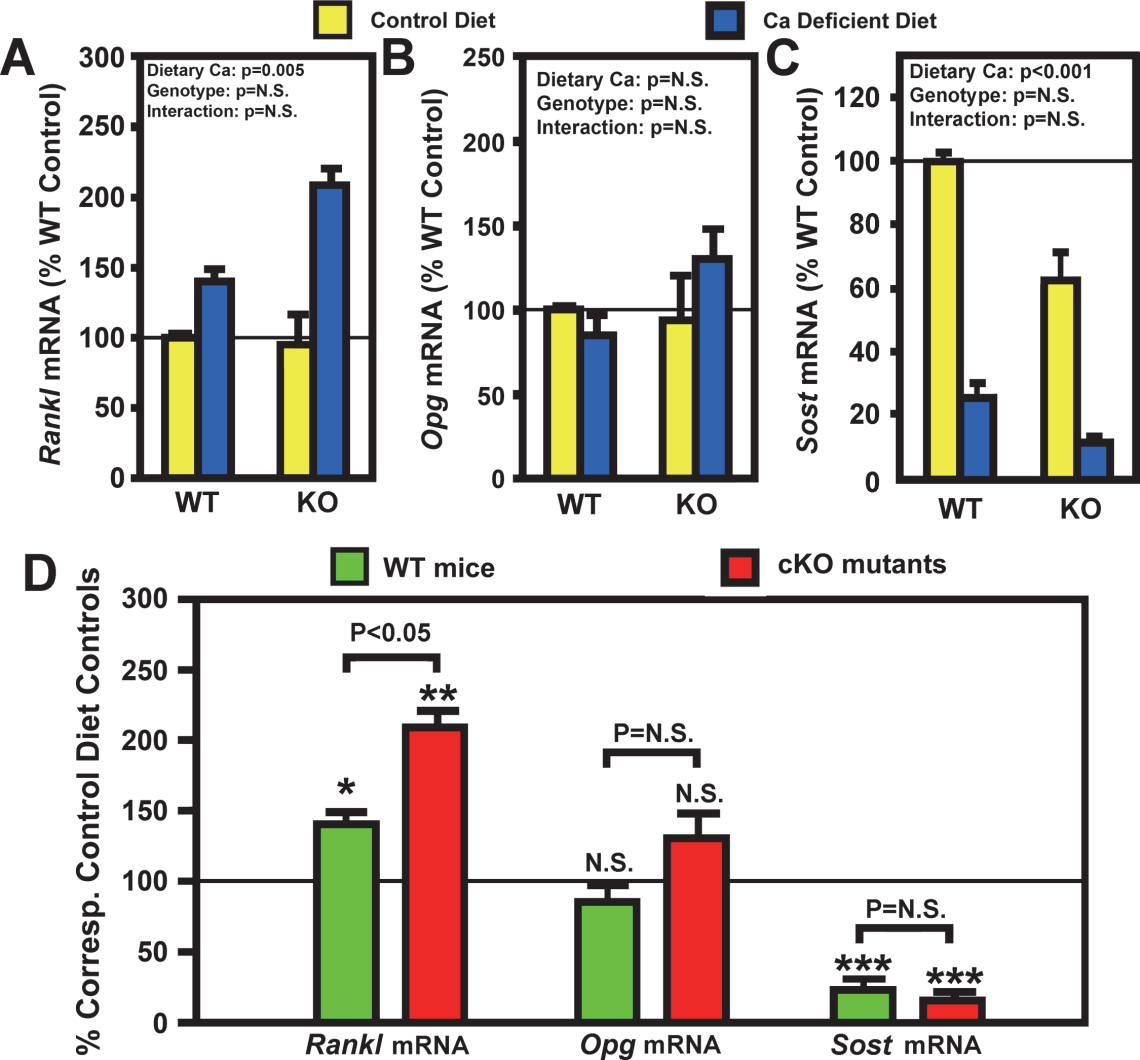
Effects of two-week dietary calcium restriction on the *Rankl* and the *Opg* mRNA levels of osteocyte *Igf1* cKO mice and those of WT mice. Total RNA was isolated from bone powders of entire femur of cKO and WT mice after two weeks on calcium-containing or-deficient diet (n = 4 animals per group). mRNA levels of *Rankl* and *Opg* by real-time RT-PCR, and are shown as mean ± SEM. A: the *Rankl* mRNA levels as relative percentage of that in WT control (i.e., on calcium-containing diet); B is the *Opg* mRNA levels as percentage of that in WT control; and B: comparison of the effect of calcium deficiency on *Rankl* and *Opg* mRNA in either mouse strain. *P<0.05; **P<0.01; and ***P<0.001. P = N.S. (not significant), where P>0.05.

RANKL and its decoy receptor, osteoprotegerin (OPG), are key regulators of osteoclast differentiation and activity. To determine the potential mechanism for the small enhanced bone resorption response in cKO mice, we measured the relative levels of *Rankl* and *Opg* mRNA in the bone (including bone marrow) of cKO mutants and WT control mice after two weeks of dietary calcium restriction. Consistent with our previous report [26], the basal *Rankl* (Fig. 4A) and *Opg* (Fig. 4B) mRNA levels in bones of osteocyte *Igf1* cKO mice were not different from those of age- and gender-matched WT control mice. Importantly, the calcium depletion significantly increased *Rankl* mRNA, but not *Opg* mRNA, in bones of both mouse strains (Fig. 4C). When each mRNA species was normalized against each respective control, the calcium deficient-induced upregulation of *Rankl* mRNA was significantly larger (P<0.05) in cKO mutants than in WT controls (Fig. 4D). There was no significant difference in the response of the *Opg* mRNA expression to calcium depletion in either mouse strain. Therefore, the effect of calcium depletion on the ratio of *Rankl* mRNA/*Opg* mRNA, which is more important than the *Rankl* expression level alone in regulating osteoclast differentiation and activity, should also be similar to that on the *Rankl* mRNA. Accordingly, these findings raise the possibility that the apparent enhanced bone resorption response to calcium depletion in osteocyte *Igf1* cKO mice may in part be due to a larger increase in RANKL expression in response to calcium depletion in cKO mutants compared to WT mice.

Previous studies have reported that calcium depletion also led to inhibition of bone formation in rodents [28,35]. Consistent with these findings, the two weeks dietary calcium depletion significantly reduced trabecular BFR in WT mice by 45% (1.84 ± 0.24 vs. 3.90 ± 0.57 mm^2^ × 10^3^/day, n = 5–6 mice per group, p<0.01). We did not measure the effect of calcium depletion on trabecular BFR in our osteocyte *Igf1* cKO mutants, but it is likely that calcium depletion may also suppress trabecular BFR in these cKO mutants. Because SOST is a major local paracrine factor produced by osteocytes to regulate bone formation [12], we measured basal bone levels of *Sost* mRNA and also assessed the effects of calcium depletion on the bone content of *Sost* mRNA level in both groups of mice. The osteocyte *Igf1* cKO mutants had 40% lower basal *Sost* mRNA levels in the bone than that corresponding WT mice (Fig. 4C), suggesting that osteocyte-derived IGF-I may have a positive regulatory function on the osteocyte expression of SOST. However, because cKO mutants had higher basal plasma PTH level (Table 2), which is a negative regulator of *Sost* expression in the bone [38], an alternative possibility is that the lower basal *Sost* mRNA level in cKO mutants was a direct consequence of the elevated basal plasma PTH level. Importantly, calcium depletion drastically reduced the *Sost* mRNA content by 80–90% in bones of both mouse strains (Fig. 4C). It is conceivable that the large increase in plasma PTH in response to calcium depletion (Table 2 and Fig. 2B) may have a key role in the observed drastic reduction in the bone content of SOST in both groups of mice (Fig. 4C&D). Nevertheless, when the *Sost* mRNA level in the calcium depletion group of each mouse strain was normalized against respective control group, there was no significant difference in the suppressive effect of calcium depletion on *Sost* mRNA expression in either mouse strain (Fig. 4D). It is noteworthy that despite the dramatic reduction in SOST with calcium depletion, there was still a highly significant reduction in BFR in both groups of mice.

### Effects of deficiency of osteocyte-derived IGF-I on calcium repletion-induced bone formation

**Figure 5:**
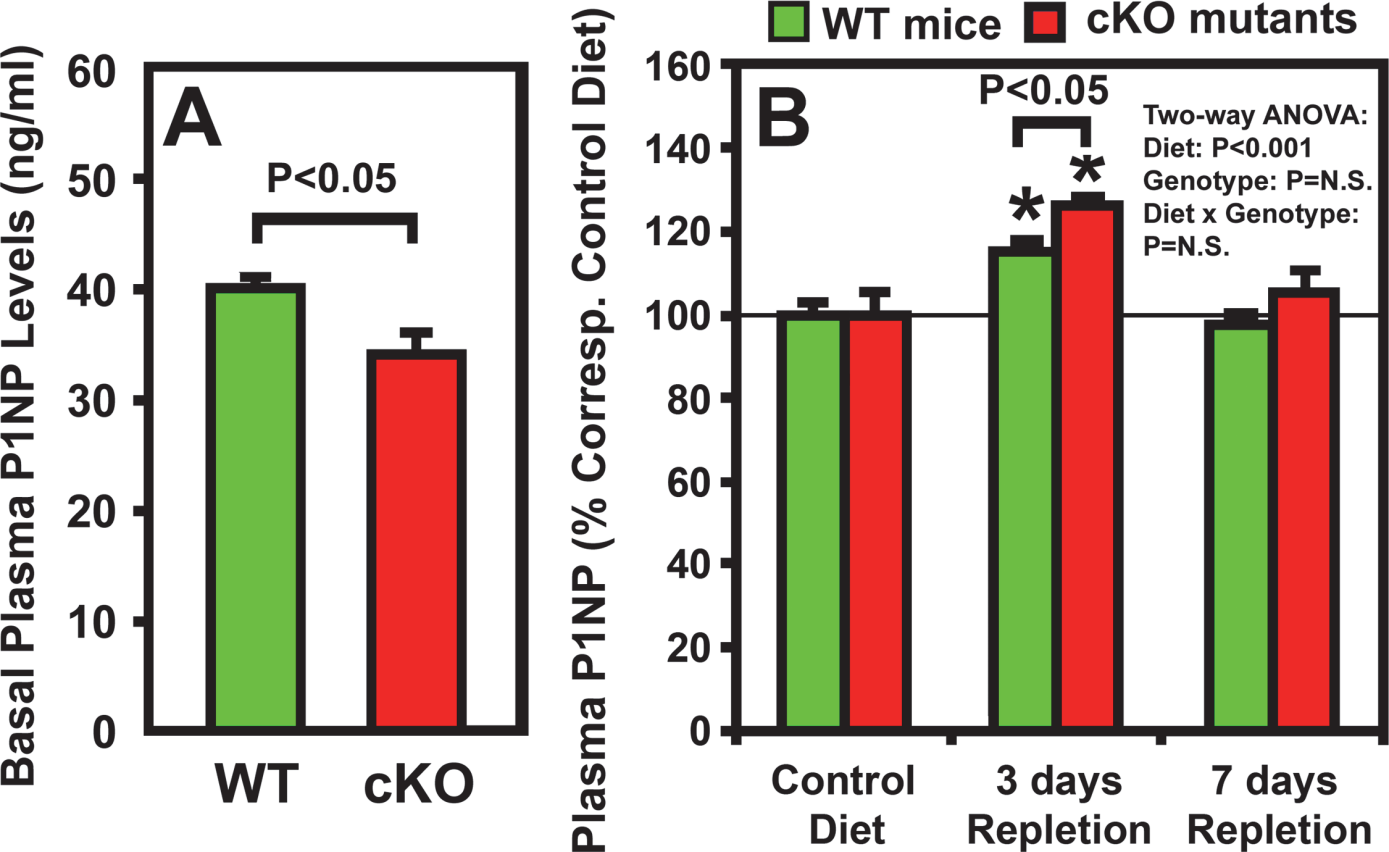
Effects of one-week of dietary calcium repletion on plasma P1NP level in osteocyte *Igf1* cKO mutants and in corresponding WT mice. Four-week-old osteocyte *Igf1* cKO mice (n = 8) and age- and gender-matched WT control mice (n = 10) were fed either the calcium-deficient or the calcium-containing diet for two weeks followed by 1 week calcium-containing diet. Plasma samples were obtained at the beginning of the experiment (basal), and after three days and 7 days of calcium repletion. Results are shown as mean ± SEM. A shows the actual basal level of P1NP; and B shows the effect of the dietary calcium repletion in the two strains, which is reported as relative percentage changes of the basal level of WT control mice fed the calcium-containing diet for east indicated test time period. *P<0.05.

Previous studies in growing rodents have indicated that dietary calcium repletion would rapidly stimulate bone formation to replace the bone mass lost during calcium depletion [4,5,31,32]. Thus, we next sought to determine whether deficient *Igf1* expression in osteocytes would alter the bone repletion response. We compared plasma level of P1NP (a bone formation biomarker) of osteocyte *Igf1* cKO mice with that of corresponding WT control mice after 3 or 7 days of calcium repletion. The cKO mutants not only have 28% lower basal plasma CTx level than WT control mice (Fig. 3A), but also have 15% lower basal plasma P1NP level than corresponding WT mice (Fig. 5A). These findings are consistent with our previous conclusion that basal bone remodeling rate in osteocyte *Igf1* cKO mice is lower than WT mice during developmental growth [26]. The plasma P1NP level in both cKO mutants and WT control mice was each significantly increased at day 3 of dietary calcium repletion (Fig. 5B). In addition, the increase in plasma P1NP was slightly but significantly (P<0.05) greater in cKO mutants than that in WT control mice. There was no significant genotype effect (i.e., effects that were due to deficient osteocyte-derived IGF-I) nor was there an interaction between dietary calcium and osteocyte-derived IGF-I deficiency on plasma P1NP level (Fig. 5B). The plasma P1NP level of either mouse strain was reverted back toward respective basal level at the end of the 7 days calcium repletion.

**Figure 6:**
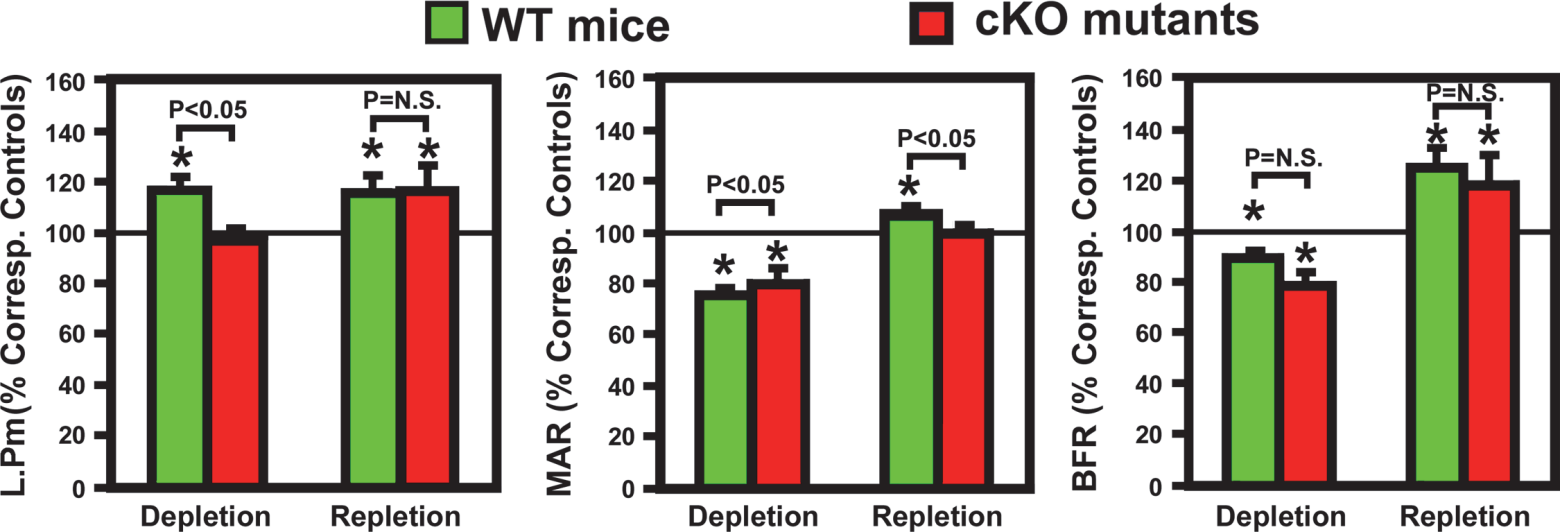
Effects of dietary calcium depletion and subsequent repletion on dynamic histomorphometric parameters of bone formation in femurs of osteocyte *Igf1* cKO mutants and corresponding WT mice. Left panel: the bone mineralizing surface, shown as relative percentage of TLS of corresponding controls (mice of corresponding mouse strain fed the calcium-containing diet throughout); middle panel: the bone mineralization apposition rate; and right panel: the calculated bone formation rate per bone surface. Results are shown as mean ± SEM. The number of mice per group is shown in Table 4. *P<0.05.

We next determined endocortical bone formation histomorphometric parameters at the mid-point of femurs of both mouse strains. Table 4 shows that basal endosteal BFR of cKO mutants was significantly lower (by 26%, P<0.05) than that of WT control mice. The lower basal endosteal BFR in cKO mutants remained significant even after normalization against bone surface length (BFR/B.Pm). The basal tetracycline labeling surface length (L.Pm) and basal mineral apposition rate (MAR) were also lower by 10% and 17%, respectively, but these differences did not reach statistically significant levels. The two-week calcium depletion significantly reduced endosteal MAR and BFR in both mouse strains (Table 4). L.Pm was increased (by 17%, P<005) in WT control mice but not in cKO mutants (Table 4 and Fig. 6). In addition, the calcium depletion also reduced periosteal BFR in both WT mice (reduced to 57±4% of that of corresponding WT control mice, n = 6 per group, p<0.05) and cKO mutants (reduced to 39±6% of that of corresponding cKO control mice, n = 5 mice per group, p<0.05), indicating that dietary calcium deprivation also suppressed bone formation at the periosteal bone surface.

**Table 4:** Effects of two weeks of dietary calcium depletion followed by one week of dietary calcium repletion on dynamic bone formation histomorphometric parameters of at the endosteal surface of the secondary spongiosia of the distal femur.

**Parameter**	**Control Diet for Depletion**		**Ca Depletion**		**Control Diet for Repletion**		**Ca Repletion**		**Two-way ANOVA**		
	WT (n = 7–8)	*Igf1* cKO (n = 7–8)	WT (n = 7–8)	*Igf1* cKO (n = 7–8)	WT (n = 6)	*Igf1* cKO (n = 4)	WT (n = 5–6)	*Igf1* cKO (n = 5–6)	Diet	Gene	interaction
E. L.Pm (mm^2^)	2.49±0.07	2.24±0.10	2.91±0.12	2.17±0.10***	2.30±0.13	1.98±0.17	2.88±0.17	2.61±0.22	<0.05	P<0.001	P = N.S.
E. L.Pm/B.Pm	0.68±0.02	0.65±0.03	0.72±0.02	0.60±0.03**	0.68±0.04	0.66±0.04	0.76±0.05	0.77±0.05	<0.05	P<0.001	P = N.S.
E. MAR (μm/d)	3.14±0.24	2.62±0.23	2.38±0.08	2.09±0.15	1.44±0.13	1.15±0.07	3.37±0.07	2.61±0.08***	<0.001	P<0.001	P = N.S.
E. BFR (mm^2^×10^3^/d)	7.76±0.57	5.77±0.37*	6.95±0.18	4.56±0.27**	7.21±0.67	5.73±0.36	9.70±0.58	6.83±0.64*	<0.001	P<0.001	P = N.S.
E. BFR/B.Pm (mm^2^×10^3^/mm/d)	2.10±0.11	1.68±0.08*	1.72±0.03	1.27±0.06*	3.27±0.23	2.26±0.14**	2.58±0.16	2.00±0.16	<0.001	P<0.001	P = N.S.

E = endosteal; L.Pm = tetracycline labeling surface (dL.Pm + ½ sL.Pm); B.Pm = bone surface; MAR = mineral apposition rate; BFR = bone formation rate.

P = N.S. (not significant), when P>0.05.

* P<0.05; ** P<0.01; *** P<0.001, when compared to respective control mice on the control diet.

The one-week calcium repletion increased MAR by 40% in the WT and 25% in cKO mice. Calcium repletion resulted in 20% increase in L.Pm (P = 0.07) and 28% increase in L.Pm/B.Pm (P<0.05) in cKO mutants but had no effect on L.Pm in WT mice. When the bone formation parameters were normalized against each respective basal level (Fig. 6), the calcium repletion-induced increases in these bone formation parameters, with the exception of MAR, did not differ significantly between the two mouse strains. Thus, deficient IGF-I expression in osteocytes does not have a significant impact on the bone repletion response.

### Effects of deficiency in osteocyte-derived IGF-I on changes in bone size and bone mineral density (BMD) in response to calcium depletion/repletion

**Figure 7:**
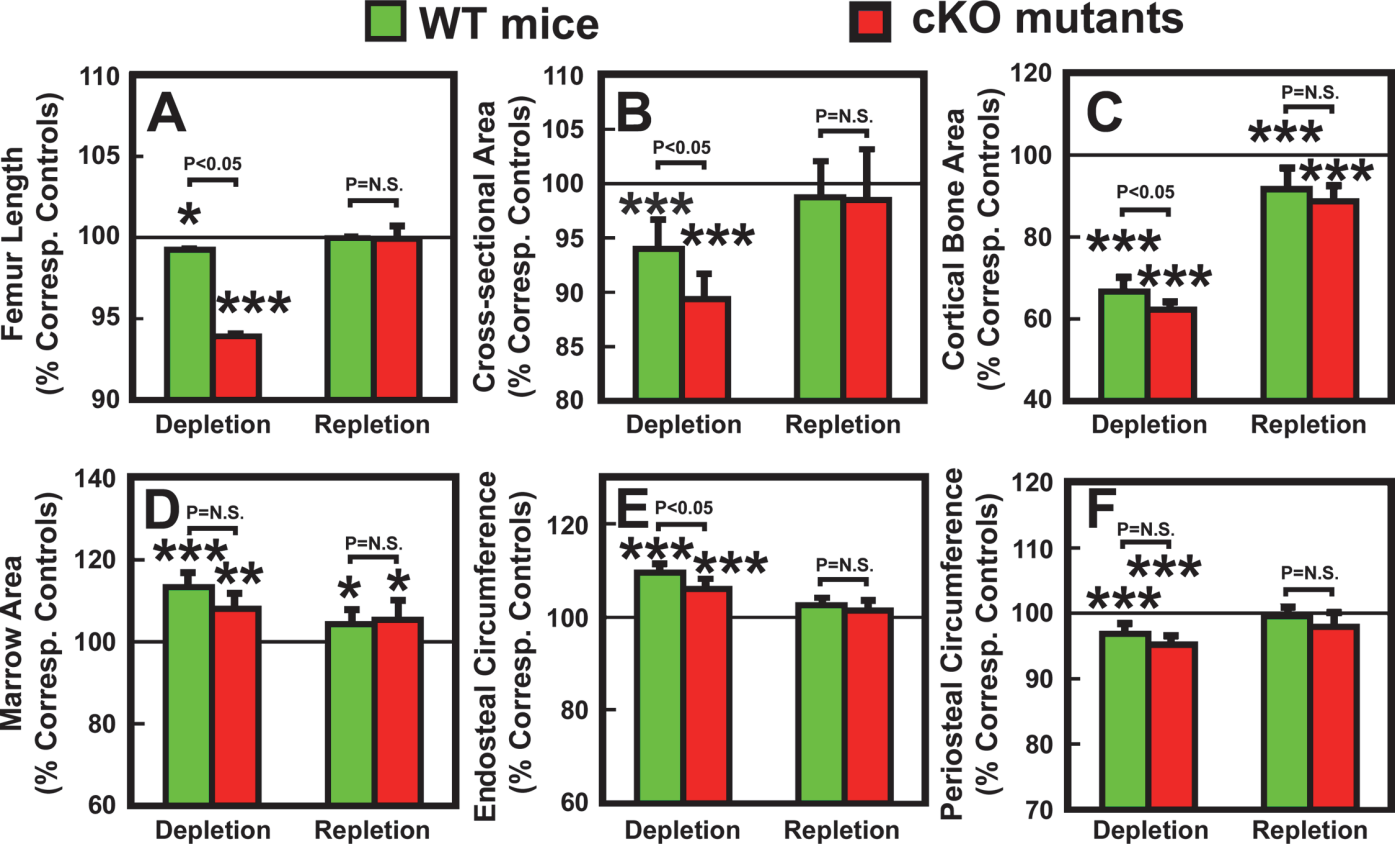
Effects of calcium depletion and repletion on bone size parameters in femurs of osteocyte *Igf1* cKO mutants and corresponding WT mice. With the exception of the femur length, all bone size parameters were measured at the mid-diaphysis of femur by pQCT. Results are shown as relative percentage of corresponding control mice (mean ± S.E.M.). The number of mice per group is shown in Table 6. *P<0.05; and ***P<0.001.

To determine if deficient expression of osteocyte-derived IGF-I would have an effect on bone size and BMD (two key determinants of bone strength) in response to bone depletion/repletion, we compared the consequence of the two-week calcium depletion and the one-week repletion on the various bone size, bone mineral content (BMC), and BMD parameters in femurs of osteocyte *Igf1* cKO mutants with those of WT control mice. Consistent with our previous report that deficient osteocyte *Igf1* expression impaired developmental bone growth [26], the cKO mutants had ~3% shorter femur, 13% decrease in total cross-sectional bone area, 12% reduction in cortical area, 13% decrease in marrow area, and 7–8% each decreases in endosteal and periosteal circumferences compared to WT control mice (Table 5). The two-week calcium depletion reduced significantly total cross-section area, cortical area, and periosteal circumference, and increased marrow area and endosteal circumference in both mouse strains. The one-week repletion almost completely reversed the calcium depletion-induced effects on these bone size parameters and returned them to levels approaching those of the corresponding control mice of each mouse strain (Table 5). To evaluate whether deficient *Igf1* expression in osteocytes would affect calcium depletion/repletion-induced effects on bone length and size, we compared the relative % change in each parameter from each respective control basal value between cKO mutants and WT control mice. The effects of two-week calcium depletion on each parameter, other than periosteal circumference, were significantly (p<0.05) greater in cKO mutants than in WT mice (Fig. 7). In contrast, the effects of one-week calcium depletion on each test parameter were not significantly different between the two mouse strains. Thus, it is likely that the significant genotype effect (effects due to deficient *Igf1* expression in osteocytes) indicated by two-way ANOVA (Table 5) on each parameter was due primarily to differences seen during depletion but not during repletion.

**Table 5:** Comparison of cortical cross-sectional bone parameters at the mid-shaft of femurs between WT and *Igf1* cKO mice after two-week dietary calcium depletion and/or after one-week dietary calcium repletion.

**Parameter**	**Control for Depletion^#^**		**Depletion**		**Control for Repletion^#^**		**Repletion**		**Two-way ANOVA**		
	**WT (n = 5)**	**cKO (n = 5)**	**WT (n = 8)**	**cKO (n = 7)**	**WT (n = 7)**	**cKO (n = 8)**	**WT (n = 5)**	**cKO (n = 6)**	**Diet**	**Gene**	**Interaction**
Femur length, mm	13.4±0.1	13.1±0.1	13.3±0.1	12.3±0.1^c^	13.7±0.1	13.2±0.2	14.0±0.1	13.3±0.1^a^	P<0.001	P<0.001	P<0.01
Total X-section area, mm^2^	1.41±0.01	1.15±0.04^c^	1.42±0.04	1.17±0.03^a^	1.51±0.04	1.31±0.05^c^	1.49±0.05	1.29±0.06^c^	P<0.05	P<0.001	P = N.S.
Cortical area, mm^2^	0.58±0.01	0.48±0.03^c^	0.40±0.02	0.33±0.01^c^	0.60±0.02	0.53±0.03^c^	0.55±0.03	0.47±0.02^c^	P<0.001	P<0.001	P = N.S.
Marrow area, mm^2^	0.83±0.01	0.67±0.04^c^	1.02±0.03	0.84±0.03^a^	0.90±0.03	0.78±0.04^c^	0.94±0.03	0.82±0.04^c^	P<0.05	P<0.001	P = N.S.
Endosteal circumf, mm	3.55±0.01	3.19±0.10^c^	4.03±0.07	3.63±0.07^b^	3.68±0.09	3.42±0.07	3.77±0.06	3.46±0.08^c^	P = 0.002	P<0.001	P = N.S.
Periosteal circumf, mm	4.52±0.02	4.10±0.12^c^	4.53±0.07	4.12±0.06^c^	4.68±0.07	4.33±0.09	4.65±0.07	4.24±0.09^c^	P = N.S.	P<0.001	P = N.S.

Results are shown as mean ± SEM.

^#^ The control group are mice receiving normal calcium-containing diet throughout.

^a^ P<0.001; ^b^ P<0.01; ^c^ P<0.05; when compared to corresponding WT littermates.

P = N.S., not significant, when P>0.05.

**Table 6:** Comparison of bone mineral content (BMC) and density (BMD) of the mid-point femurs between WT and *igf1* KO mice during calcium depletion or repletion.

**Parameters**	**Control for Depletion**		**Depletion**		**Control for Repletion**		**Repletion**		**Two-way ANOVA**		
	**WT (n = 18)**	**cKO (n = 15)**	**WT (n = 10)**	**cKO (n = 10)**	**WT (n = 5)**	**cKO (n = 6)**	**WT (n = 12)**	**cKO (n = 11)**	**Diet**	**Gene**	**Interaction**
total BMC (mg)	0.78±0.02	0.70±0.03^c^	0.56±0.02	0.46±0.03^b^	0.81±0.04	0.66±0.06	0.76±0.02	0.62±0.02^a^	P<0.001	P<0.001	P = N.S.
cort BMC (mg)	0.57±0.02	0.50±0.02^c^	0.28±0.03	0.21±0.02	0.60±0.04	0.49±0.05	0.53±0.02	0.41±0.02^a^	P<0.001	P<0.001	P = N.S.
trab BMC (mg)*	0.17±0.01	0.16±0.01	0.22±0.01	0.17±0.01^b^	0.16±0.01	0.11±0.02^c^	0.18±0.01	0.16±0.01^c^	P = 0.001	P = 0.001	P = 0.06
total BMD (mg/mm^3^)	486±6	489±6	345±11	341±8	510±11	537±7	442±6	442±8	P<0.001	P = N.S.	P = N.S.
cort BMD (mg/mm^3^)	949±6	941±9	794±11	765±8^c^	969±13	976±16	907±8	877±11^c^	P<0.001	P = 0.018	P = N.S.
Trab BMD (mg/mm^3^)*	140±4	145±4	150±3	140±4^a^	145±5	150±6	140±4	149±3	P = N.S.	P = N.S.	P = 0.059

Data are shown as Mean ± SEM.

^#^ The control group are mice receiving normal calcium-containing diet throughout.

^a^ P<0.001; ^b^ P<0.01; ^c^ P<0.05; when compared to corresponding WT littermates.

P = N.S., not significant, when P>0.05.

* Measured at metaphysis area immediately from secondary spongiosia.

**Figure 8:**
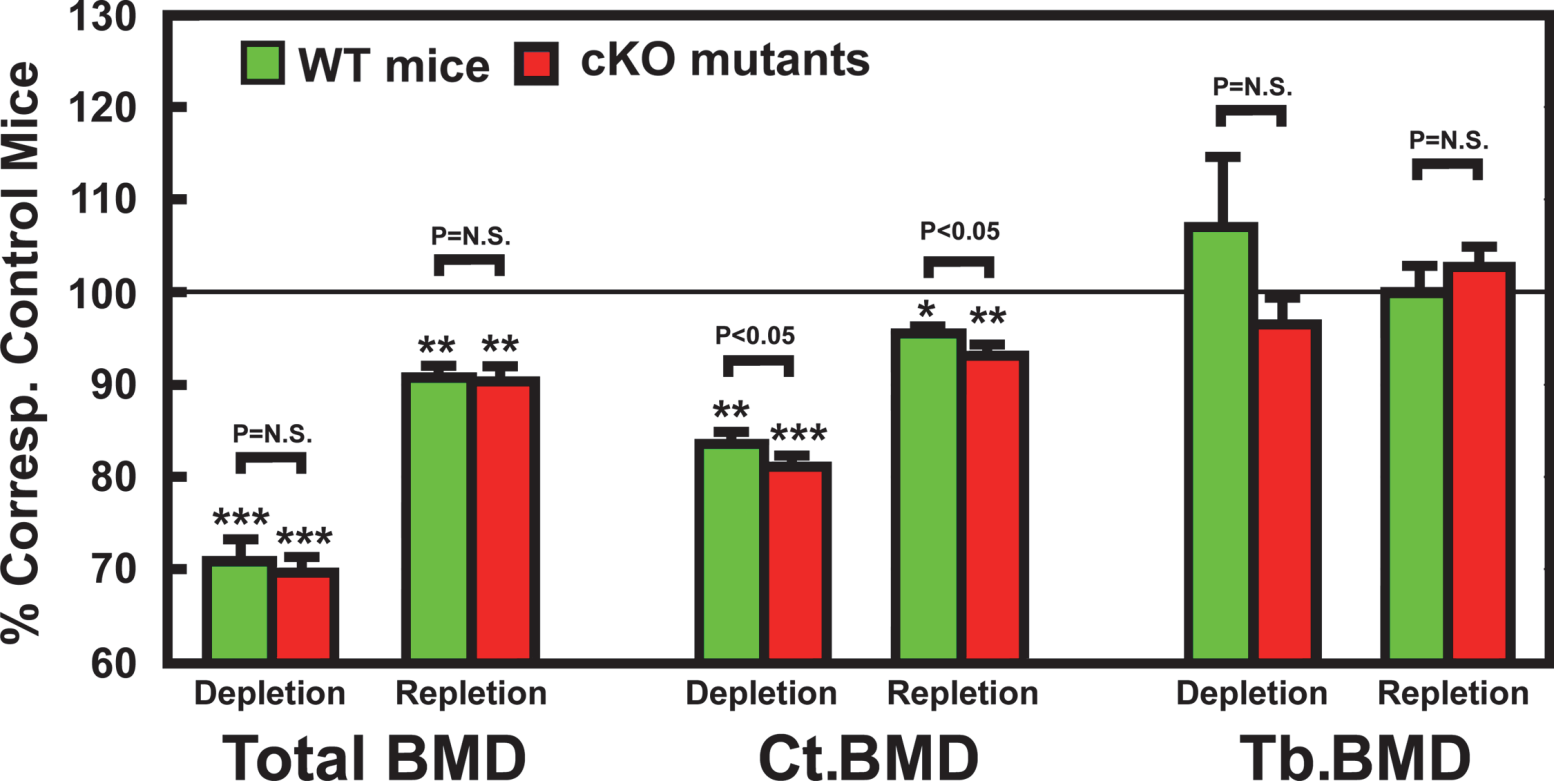
Effects of calcium depletion and repletion on BMD parameters in femurs of osteocyte *Igf1* cKO mutants and corresponding WT mice. BMD parameters were determined by pQCT. Total BMD was performed on the entire bone; cortical BMD was measured at the mid-diaphysis; and Trabecular BMD was determined at the site corresponding to secondary spongiosa. Results are shown as relative percentage of corresponding control mice (mean ± S.E.M.). The number of mice per group is shown in Table 6. *P<0.05; and ***P<0.001.

The effects of calcium depletion/repletion on BMC and BMD are summarized in Table 6. Consistent with our previous report [26], femurs of cKO mutant mice fed a normal calcium diet throughout had ~10% reduction each in total and cortical BMC, as well as in total, trabecular, and cortical BMDs, when compared to WT control mice. The two-week calcium depletion reduced, whereas the one-week calcium repletion increased, total and cortical (but not trabecular) BMC and BMD in both mouse strains (Table 6). When BMC and BMD values are normalized against each corresponding basal value, the depletion-mediated reduction in cortical BMC and BMD (but not depletion-related decreases in total or trabecular BMC and BMD) in cKO mutants was significantly (P<0.05) bigger than those in WT mice (Fig. 8). However, there was no significant mouse strain-related difference in the repletion-induced restoration of BMC and BMD in either cortical or trabecular bones (Fig. 8). The fact that deficient expression of *Igf1* in osteocytes did not have an effect on the repletion-induced restoration of BMC and BMD suggests that osteocyte-derived IGF-I is probably not a key regulator of the bone repletion-mediated bone formation.

## Discussion

We have recently reported that disruption of *Igf1* gene in osteocytes impaired developmental bone growth that was associated with suppression in bone remodeling [26]. Because the bone remodeling process is mechanistically similar to the bone depletion/repletion response [4], we had originally predicted that conditional deletion of *Igf1* in osteocytes would also negatively affect the bone depletion/repletion process. To our surprise, disruption of *Igf1* expression in osteocytes did not have significant negative effects on bone depletion or repletion. This conclusion was based on two key findings: First, two weeks of dietary calcium restriction induced hypocalcemia and the associated secondary hyperparathyroidism to similar levels in both osteocyte *Igf1* cKO mutant mice and age- and gender-matched WT control mice (Table 2 and Fig. 2). These effects led to an increase in bone resorption [as reflected by the significant increases in plasma CTx and bone resorption histomorphometry parameters (Fig. 3)], which in turn resulted in significant loss of BMC (Table 4) and BMD (Table 4 and Fig. 7) in both mouse strains. In addition, the calcium depletion suppressed BFR in both cKO mutants and WT mice to the similar extent (Fig. 6). Second, dietary calcium repletion acutely increased bone formation [as indicated by an elevated level of plasma P1NP after 3 days of dietary calcium repletion (Fig. 5B) and increases in MAR and BFR (Fig. 6)] in both cKO mutants and WT control mice. This repletion-induced increase in bone formation returned the BMC and BMD level of each mouse strain almost completely back to their respective basal levels (Fig. 7). Thus, inasmuch as osteocyte-derived IGF-I is a key positive regulator of bone remodeling, it does not appear that osteocyte-derived IGF-I is an important regulator of the bone response to dietary calcium depletion and repletion.

Interestingly, conditional disruption of *Igf1* in osteocytes not only did not impair, but in fact slightly and significantly enhanced the calcium depletion-induced bone resorption response. Accordingly, the osteocyte *Igf1* cKO mutant mice exhibited significantly greater (by 150–200%) increases in plasma CTx and in N.Oc/B.Pm, along with significant reductions in Tb.N (Fig. 3) and cortical BMD (Fig. 7), in response to the two weeks of calcium depletion than corresponding WT control mice. The larger bone resorption response to calcium depletion in cKO mutants is consistent with the previous report that calcium depletion exerted greater increases in bone resorption and bone loss in *Igf1* global KO mice [30]. The molecular mechanism responsible for the larger bone resorption response to calcium depletion in osteocyte *Igf1* cKO mice is unclear. However, the dietary calcium restriction increased *Rankl* mRNA levels in the bone in both mouse strains, but the calcium deficiency-induced increase in *Rankl* mRNA (but not *Opg* mRNA) in bones of cKO mutants was almost 2-fold of that in bones of WT control mice (Fig. 4). Because the effect of the same dietary calcium deprivation on the bone levels of *Opg* mRNA, the soluble decoy receptor of RANKL, in cKO mutants was not significantly different from that in WT mice, it is likely that the larger bone resorption response to calcium depletion in osteocyte *Igf1* cKO mice is in part due to bigger calcium depletion-induced local RANKL production in bone. These results would suggest that osteocyte-derived IGF-I is a negative regulator of the calcium-induced bone resorption and that its inhibitory action is mediated in part through suppression of RANKL (without affecting OPG) in the bone. These findings are unexpected, since previous studies reported that deficiency in IGF-I in *Igf1* global KO mice reduced osteoclastogenesis through suppression of *Rankl* expression in the bone [21]. In addition, the finding that our osteocyte *Igf1* cKO mice exhibited a larger response in osteoclastogenesis and bone resorption to calcium depletion contradicts the previous findings that the IGF-I derived from osteoblasts [39] or other cell types [21] stimulates osteoclastogenesis (and thereby bone resorption) through upregulation of the *Rankl* expression. It is possible that IGF-I under different context (e.g., derived from osteoblasts vs. from osteocytes) may have different actions on osteoclastogenesis and bone resorption. Thus, understanding of the exact molecular mechanism that directs the negative regulatory action of osteocyte-derived IGF-I on the calcium depletion-induced *Rankl* expression and osteoclastogenesis is important, but it would require much additional studies.

The more intriguing finding of this study perhaps is the lack of an adverse effect of deficient expression of osteocyte-derived IGF-I on the bone repletion process. Accordingly, the one-week dietary calcium repletion in cKO mutants appeared to be equally effective in increasing the plasma (Fig. 5) and dynamic bone histomorphometric (Fig. 6) bone formation parameters as WT littermates. The relative extent of the return of bone mass (Fig. 7) and BMD (Fig. 8) after the one-week calcium repletion was also similar in both mouse strains. The lack of a negative effect of deficient expression of osteocyte-derived IGF-I on the bone repletion process is surprising, especially in light of our recent discovery that the two-week regimen of four-point bending mechanical loading exercise failed to elicit the bone formation response in these same osteocyte *Igf1* cKO mutants [27], which implicates an essential role of osteocyte-derived IGF-I in loading-mediated bone formation. The bone repletion response and the anabolic response to mechanical loading are both important physiological guardians of maintenance of bone mass and strength. Therefore, our findings that target disruption of *Igf1* gene in osteocytes had no discernable negative effects on the bone depletion process even though this osteocytic growth factor is an essential mediator of mechanosensitivity in the bone suggest a dichotomy between the mechanisms necessary for the osteogenic response to loading and the regulatory hormonal and anabolic skeletal repletion following low dietary calcium challenge.

**Figure 9:**
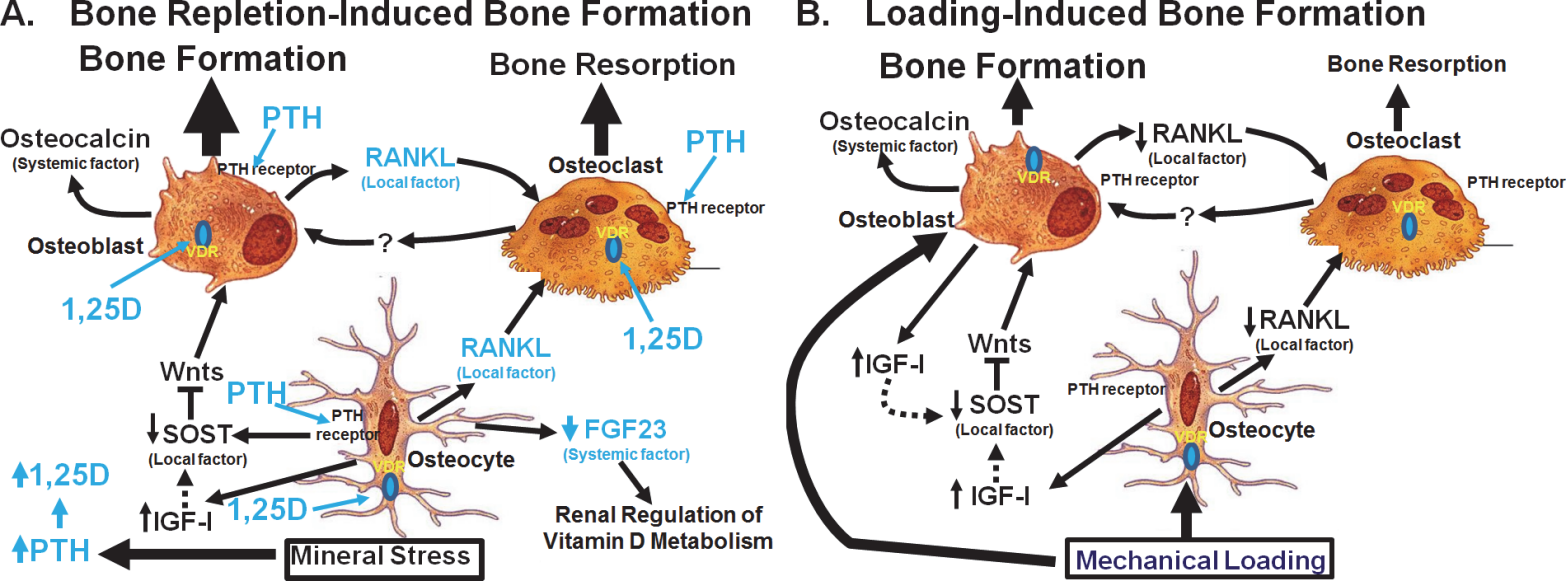
Proposed cellular mechanisms contribute to bone repletion-induced bone formation (A) or to loading-induced bone formation (B). Please refer to text for details.

The mechanistic reason(s) contributing to the lack of a significant regulatory role of osteocyte-derived IGF-I in bone repletion after a severe calcium stress is unclear. In this regard, we have proposed a model (Fig. 9A) that may provide mechanistic insights into why deficient osteocyte-derived IGF-I did not have significant effects on the bone repletion process. In this model, it is well established that the calcium stress caused secondary hyperparathyroidism and elevated circulating levels of 1,25(OH)_2_D_3_ [3,28,31,33,34]. Accordingly, we have demonstrated that calcium depletion markedly increased plasma PTH levels in both cKO mutants and WT mice. Although we did not measure plasma levels of 1,25(OH)_2_D_3_ in this study, it is reasonable to suspect that the elevated plasma PTH, which is a potent activator of renal production of 1,25(OH)_2_D_3_, as well as the large reduction in plasma FGF23, which is a potent suppressor of renal production of 1,25(OH)_2_D_3_ [15,16], occurred during calcium depletion would result in a large increase in the circulating level of 1,25(OH)_2_D_3_ in both cKO mutants and WT mice. Both of these systemic hormones are generally accepted to be the primary driving force for the bone resorption response during the calcium stress [4,5,28,31], since these systemic hormones, especially PTH, are potent stimulators of bone turnover. Our previous studies [26] have implicated that osteocyte-derived IGF-I plays a regulatory role in the local bone remodeling process, as deficient osteocyte-derived IGF-I significantly suppressed bone turnover in growing mice. However, we postulate that the local effect of deficient osteocyte-derived IGF-I on bone remodeling might have been masked by the huge systematic effects of hyperparathyroidism on bone turnover and bone loss. Accordingly, it is conceivable that osteocyte-derived IGF-I is a local regulator of bone remodeling and does not participate in systemic regulation of bone turnover, which is mediated largely through systemic effectors, such as PTH and 1,25(OH)_2_D_3_. In any event, all three major cell types (osteocytes, osteoblasts, and osteoclasts) in the bone express receptors for PTH and for 1,25(OH)_2_D_3_ (VDR) [40,41,42,43], and both PTH and 1,25D are not only activators of osteoblasts, but can also activate osteoclast differentiation. We postulate that under the condition of calcium deficiency, the two systemic hormones favor bone resorption over bone formation through a poorly understood mechanism [5,28]. We also speculate that the reduced sensitivity of osteoblasts to PTH and 1,25(OH)_2_D_3_ under conditions of insufficient bone minerals is an important physiological protective mechanism, in that bone minerals are used for the maintenance of bone mineral homeostasis rather than for mineralization of newly formed bone matrix. However, when the calcium deficiency is corrected by repletion, the rapid increase in the circulating calcium would rapidly induce osteoclast apoptosis [4,32] to inhibit further bone resorption, but the circulating levels of PTH and 1,25(OH)_2_D_3_ during the early phase of calcium repletion remain elevated [3,4,31]. Accordingly, these two systemic hormones, in the presence of sufficient calcium, would favor bone formation over resorption, leading to rapid increases in bone formation during repletion (Fig. 9A).

This model may also account for the apparent dichotomy between the mechanisms necessary for anabolic responses to mechanical loading and the regulatory hormonal and anabolic skeletal repletion following the low dietary calcium challenge (Fig. 9B). Accordingly, we postulate that, upon mechanical loading, osteocytes (the primary mechanosensing cell type in the bone [8]) reduce production and release of SOST (an inhibitor of the Wnt signaling). This would lead to activation of canonical Wnt signaling in nearby osteoblasts, resulting in subsequent increases in local bone formation [8,12]. The loading strains also enhance local production and release of IGF-I from osteoblasts and also upregulate IGF receptors in osteoblasts [44]. These actions then further enhance the osteogenic response to loading. The mechanical loading also reduces osteocytic RANKL production, which leads to reduction in osteoclast differentiation and resorption activity [45]. We postulate that the loading-mediated activation of osteoblasts also reduce osteoblastic production of RANKL, leading to further suppression in osteoclast differentiation, activity, and thereby bone resorption. Consequently, the overall effect of mechanical loading is activation of osteoblasts and bone formation and suppression of osteoclastic resorption. Because osteocyte-derived IGF-I is essential and also required for these loading-induced anabolic effects, disruption of *Igf1* in osteocytes would abolish the loading-induced anabolic effects in the bone [27]. We believe that the local loading-induced bone formation process shown in Fig. 9B remains operational during bone depletion/repletion, but the magnitude of these local bone effects is much lower than that of the systemic-induced bone effects. However, we should note that in the case of calcium repletion, the SOST levels in the bone following calcium depletion are likely to have already been very low (Fig. 4 C&D). The anticipated further down-regulation of SOST in response to calcium repletion would not be expected to result in large increase in bone formation. Accordingly, we postulate that unlike the loading-induced bone formation, down-regulation of SOST expression may not be the only (or the key) mechanism contributed to the calcium repletion-induced bone formation. Consequently, the loss of the loading-dependent local bone formation due to deficient expression of osteocyte-derived IGF-I would only have marginal effects on the bone repletion process. We should emphasize that evidence supporting this model is largely circumstantial, but nevertheless, it is an interesting model and is worthy of further investigation.

In summary, this study has demonstrated for the first time that although osteocyte-derived IGF-I is essential in determining the bone mechanosensitivity [27], this osteocyte-derived bone growth factor does not play a major role in the regulation of bone repletion response. Consequently, there is a dichotomy between the mechanisms necessary for anabolic responses to mechanical loading and the regulatory hormonal and anabolic skeletal repletion following low dietary calcium challenge. It would be important to understand not only the molecular mechanism by which it determines the osteogenic response to loading but also the mechanism that regulates bone repletion in order to have a more complete overall understanding of how bone regeneration is regulated.
